# Autoimmune inflammatory disorders, systemic corticosteroids and pneumocystis pneumonia: A strategy for prevention

**DOI:** 10.1186/1471-2334-4-42

**Published:** 2004-10-16

**Authors:** Evin Sowden, Andrew J Carmichael

**Affiliations:** 1 Department of Dermatology, The James Cook University Hospital, Marton Road, Middlesbrough TS4 3BW UK

## Abstract

**Background:**

Pneumocystis pneumonia (PCP) is an increasing problem amongst patients on immunosuppression with autoimmune inflammatory disorders (AID). The disease presents acutely and its diagnosis requires bronchoalveolar lavage in most cases. Despite treatment with intravenous antibiotics, PCP carries a worse prognosis in AID patients than HIV positive patients. The overall incidence of PCP in patients with AID remains low, although patients with Wegener's granulomatosis are at particular risk.

**Discussion:**

In adults with AID, the risk of PCP is related to treatment with systemic steroid, ill-defined individual variation in steroid sensitivity and CD4+ lymphocyte count. Rather than opting for PCP prophylaxis on the basis of disease or treatment with cyclophosphamide, we argue the case for carrying out CD4+ lymphocyte counts on selected patients as a means of identifying individuals who are most likely to benefit from PCP prophylaxis.

**Summary:**

Corticosteroids, lymphopenia and a low CD4+ count in particular, have been identified as risk factors for the development of PCP in adults with AID. Trimethoprim-sulfamethoxazole (co-trimoxazole) is an effective prophylactic agent, but indications for its use remain ill-defined. Further prospective trials are required to validate our proposed prevention strategy.

## Background

Pneumocystis carinii was identified a hundred years ago by Chagas [1] and recognised as a pathogen in marasmic children at the end of World War II [2]. The organism came to the fore again in the early 1980s when apparently healthy homosexual men developed PCP and heralded the acquired immunodeficiency syndrome (AIDS) epidemic [3]. With highly active antiretroviral therapy (HAART) and prophylactic antibiotics, attention has turned to PCP in human immunodeficiency virus (HIV) negative individuals. We recently treated a young woman with steroid-based immunosuppression for dermatomyositis. Four months after diagnosis, she was admitted acutely breathless to the intensive care unit with a presumptive diagnosis of PCP. Although the final diagnosis was rapidly progressive alveolitis related to dematomyositis, it prompted us to consider whether we should use PCP prophylaxis for selected patients receiving systemic steroids for AID. In this article we explore the background of PCP in HIV negative patients, consider the incidence of PCP in AID, discuss predisposing factors and propose a strategy for prevention.

### Epidemiology

Pneumocystis pneumonia is caused in humans by the recently renamed Pneumocystis jiroveci (Frenkel 1999), previously known as Pneumocystis carinii and now thought to be related to fungi on the basis of DNA analysis, despite morphological similarities to protozoa [4]. The organism shows significant genetic divergence and is host specific with no cross-species infectivity. There has been considerable debate about the nature of the relationship between humans and P. jiroveci. At one time it was hypothesised that infection occurred through reactivation of colonisation acquired in childhood, as specific antibody was found in seven out of eight normal adults [5]. This suggestion has been refuted by the absence of detectable P. jiroveci in bronchoalveolar lavage specimens from healthy volunteers, despite amplification by the polymerase chain reaction [6]. In addition, genotypic analysis of P. jiroveci from infected adults identified strains found in patients' place of residence, rather than their place of birth [7]. Investigation of apparent clusters has shown different genetic strains affecting most cases, indicating that transmission from affected cases to susceptible persons does not account for the majority of infections [8,9], despite the recognised transmission of Pneumocystis DNA from affected patients to their immunocompetent contact health care workers to produce colonisation [10]. As P. jiroveci has been isolated in samples of air [11] and pond water [12], it is likely that the environment represents the main source of infection for most patients. Nevertheless, isolation of known cases of PCP is advisable.

Pneumocystis pneumonia is almost always exclusive to immunocompromised hosts. Two thirds of cases occur in HIV positive patients and constitutes the initial manifestation of AIDS in 46% of these patients [13]. A third of cases arise in HIV negative patients [14], a group consisting of organ transplant recipients (0–75%), haematological malignancies (9–58%), solid organ tumours (4–17.5%) and AID, usually on immunosuppressive treatment. The latter group accounts for 13–36% of cases in HIV negative patients [15-19]. It is this group we wish to consider in more detail.

### Pathogenesis

P. jiroveci trophozoites proliferate and attach to type I alveolar pneumocytes causing desquamation, leading to a foamy eosinophilic exudate visible on hematoxylin-eosin staining and a "honey comb" appearance of lung tissue. Both antibody and cell mediated immunity have been postulated as being involved in host protection [20].

### Clinical presentation

HIV-negative patients with PCP are older (48–61 years) than HIV positive patients. Males are affected more often than females (male to female ratio 1:1.4). The clinical course is typically more acute, with an average duration of 6–13 days. The most frequent clinical symptoms are dyspnoea (63–100%), cough (55–74%), weakness (47%), loss of appetite (38%) expectoration (22–25%), sweating (19%), weight loss (13%), haemoptysis (9%) and thoracic pain (9%). Physical findings include fever >38°C (63–85%), râles (55–66%), tachycardia (25%) and tachypnoea (22%). Chest radiography may show bilateral abnormalities (68–88%), interstitial opacities (64–80%) and alveolar opacities (31–47%), but may be normal (5–7%) [14-16,18,21]. The clinical picture can be altered by the use of aerosolised pentamidine prophylaxis, resulting in extra-pulmonary disease [20]. P. jiroveci in HIV negative patients is associated with concurrent pulmonary infections in over 50% of cases. The implicated pathogens include Cytomegalovirus (35%), Candida (18%) and Mycobacterium tuberculosis, which contribute significantly to the mortality of PCP [17,18,22].

### Diagnosis

P. jiroveci infection is diagnosed in most cases by bronchoalveolar lavage, which, depending on the staining method used, has been reported to have a sensitivity of 81 to 90% and a specificity of 90 to 100% [23]. Other methods used include hypertonic saline induced sputum production, which is less sensitive, and definitive open lung biopsy. Cysts or trophozoites are morphologically identified by methenamine-silver nitrate or giemsa stains respectively. Immunospecific stains are now available and have increased the sensitivity of detection in sputum and bronchoalveolar lavage fluid [24]. The number of organisms in diagnostic specimens is high in HIV positive patients, but often low in HIV negative cases. An induced sputum sample may therefore be insufficient in the latter situation and bronchoalveolar lavage and/or transbronchial biopsy is the preferred method of investigation [20]. In patients who are at risk of PCP it is essential to have a high index of suspicion and low threshold for investigation, to allow early diagnosis and treatment. This is particularly important in those with AID, as PCP may mimic pulmonary involvement by the underlying condition, such as dermatomyositis, as exemplified by our patient who prompted this review. In SLE, 33% of patients die from infections, with 62.5% of all fatal infections being opportunistic, but only 10% of opportunistic pathogens are detected ante-mortem [25].

### Management

Two antibiotic regimes have been shown to work, co-trimoxazole and parenteral pentamidine isethionate. Efficacies are comparable, although side-effects are particularly common with co-trimoxazole in HIV associated PCP and affect up to 60% of such patients [26]. These include rash, neutropenia, gastrointestinal upset and liver enzyme disturbance. Although side-effects are less common with parenteral pentamidine, toxicity tends to be more severe and includes pancreatitis, hypoglycaemia, neutropenia, thrombocytopenia and orthostatic hypotension. Treatment with co-trimoxazole is usually given for 21 days in HIV cases and 14 days in HIV negative PCP. Co-trimoxazole is used in 90% of cases of PCP [15] and is associated with fewer reactions (15%) in HIV negative patients [22].

### Prognosis

The prognosis of HIV negative PCP is worse than for those HIV positive, with intensive care admission in 31–60%; mechanical ventilation in 14–64%; overall mortality 19–47%, rising to 50–71% on intensive care, although some series suggest an improved mortality in recent years [14-18,27]. Poor prognosis has been associated with tachypnoea, tachycardia, elevated C-reactive protein, raised lactate dehydrogenase, mechanical ventilation, and some studies suggest a correlation with previous mean steroid dose and treatment with cyclophosphamide [17,18]. The mortality rate of patients with underlying AID appears to be worse than for other HIV negative patients [15] and varies according to underlying pathology: 63% in Wegener's granulomatosis; 58% in inflammatory myopathy; 48% in polyarteritis nodosa; 31% in rheumatoid arthritis and 17% in systemic sclerosis [27]. Several case reports suggest that PCP in inflammatory myopathy may follow a fulminant course [28].

While PCP in HIV positive patients has been associated with a high incidence of relapse after successful therapy, this has not been seen in patients with AID, even without secondary prophylaxis and despite ongoing immunosuppressive treatment. This is thought to be related to better clearance of organism, confirmed by repeat bronchoalveolar lavage [22].

### Incidence

There is no specific surveillance system in the United Kingdom (UK) for PCP, other than in HIV infection. Although laboratories are invited to report isolates of P. jiroveci to the Communicable Disease Surveillance Centre, it is estimated that only a fifth of clinically diagnosed cases of PCP are reported [29]. As the laboratory-reported number of cases was 0.36 per million in 1999, we would estimate the true incidence of PCP to be approximately 1.8 per million in that year. While Pneumocystis is the second commonest reported invasive mycosis, this estimate suggests that PCP is still a rare pathogen in the UK.

The overall incidence of PCP from 1990 to 1999 has declined in the UK [29], as a result of the advent of HAART for AIDS. However, probably as a result of the use of ever more immunosuppressive therapy, the number of cases of PCP diagnosed in HIV negative patients increased throughout the 1980s and 1990s [14-16,18,27]. These two opposing trends have resulted in HIV negative patients constituting an ever increasing proportion of the total number of cases of PCP.

Attempts have been made to characterise the incidence of PCP amongst patients with AID, usually by retrospective analysis of case records. Ward & Donald's review of 223 cases of PCP in AID is the largest series and covered 2.6 million hospitalisations over the period 1983 to 1994 [27]. They found the underlying AID in this group to be SLE (42%), rheumatoid arthritis (18%), Wegener's granulomatosis (14%), inflammatory myopathy (12%), polyarteritis nodosa (9%) and systemic sclerosis (5%). An estimate of the incidence of PCP in a particular AID was derived by determining the number of cases of PCP in a particular AID per 1,000 hospitalisations with the said AID per year. The results were: 8.9/1,000 hospitalisations /year for Wegener's granulomatosis; 6.5 for polyarteritis nodosa; 2.7 for inflammatory myopathy; 1.2 for SLE; 0.8 for systemic sclerosis and 0.2 for rheumatoid arthritis. Clearly the denominator in these frequencies reflects hospital admissions per year with a particular AID. For an AID where the average annual rate of admission is less than once per year, the true incidence for that condition will be less than the rate quoted and vice versa. These frequencies, with the exception of polyarteritis nodosa, are broadly comparable with findings in other studies which report a long-term risk of PCP of 6–12% for Wegener's granulomatosis and less than 2% for other AID [14,22,30]. There are only a few isolated reports of PCP in dermatoses treated with medium-term systemic steroids such as pemphigus, pemphigoid, cutaneous necrotizing vasculitis and Behçet's syndrome [22,31].

## Discussion

Pneumocystis pneumonia in AID is unusual in the absence of steroid treatment. Corticosteroids have been recently administered in over 90% of cases in most series [14,17,18,22] and were the sole immunosuppressant in 17–28% of AID patients [18,22]. The median duration of treatment prior to the diagnosis of PCP is three to four months. Occurrence within a month of starting treatment is uncommon, with the exception of inflammatory myopathies [28,32]. Most cases have taken prednisolone in excess of 15 mg per day, or equivalent doses of corticosteroid. Notable is the profound inter-subject variation in response to standard steroid doses as measured by in vitro inhibition of lymphocyte proliferation [33], indicating that host factors are likely to have a significant, but as yet ill-defined role. Several mechanisms have been postulated to explain the role of steroids in promoting the development of P. jiroveci including CD4+ lymphocyte depletion and immune dysfunction [17,30]. Porges et al. found an association between the risk of PCP and the dose of prednisone used in SLE [32]. Similarly, Hellman et al. found an association between prednisolone dosage and risk of fatal opportunistic infection in SLE, the commonest cause of which was P. jiroveci [26]. However, other studies have failed to show an association between cumulative steroid dose and risk of PCP [34].

A number of cytotoxic and other immunosuppressive agents commonly used in the treatment of AID are frequently associated with PCP, including cyclophosphamide, azathioprine, methotrexate and ciclosporin [14]. Cyclophosphamide is routinely used in the treatment of Wegener's granulomatosis and has transformed the previous one year survival figure of 20% [35] into the present eight year survival of 80% [36]. Godeau et al. [34] showed a significant association between cyclophosphamide cumulative dose and the risk of PCP. However, this was not an independent factor in multivariate analysis when lymphopenia was taken into account [19]. In one series involving 180 patients with Wegener's granulomatosis, no cases of PCP were identified amongst patients on cytotoxic therapy alone (although the authors did not specify the numbers involved), suggesting a permissive role for corticosteroids [30].

Data on PCP associated with AID indicates lymphocytopenia (<1,000 cells/mm^3^) is almost a prerequisite, with 91% of patients exhibiting a low lymphocyte count. Fifty percent of such PCP patients have total lymphocyte counts of <400 cells/mm^3 ^[22]. The pre-treatment lymphocyte count and lymphocyte counts during the first three months of immunosuppressive treatment in Wegener's granulomatosis have been shown to be predictive for PCP in multivariate analysis. A total lymphocyte count <600 cells/mm^3 ^was recorded in ten (83%) of 12 patients with PCP, but such a low lymphocyte count was recorded in 11 (34%) of 32 with Wegener's unaffected by PCP [34]. A similar association was found in a prospective study involving patients with SLE [37]. Porges et al [32] proposed a cut off of total lymphocyte count of <350 cells/mm^3 ^which captured 4 out of 6 cases with PCP and SLE, but only 1 of 20 patients with SLE unaffected by PCP.

Information on CD4+ counts, which have been shown to be highly predictive of the risk of PCP in HIV infected individuals [38], is less well documented in AID patients. The issue was addressed by Mansharamani et al [19] who prospectively observed 171 patients in various risk categories for PCP, including 22 patients with active PCP. They found that patients who were at high risk of PCP had significantly lower CD4+ counts than patients at low risk. They noted that 91% of cases of PCP had CD4+ counts <300 cells/mm^3 ^at the time of diagnosis. Their findings are echoed by an increased risk of respiratory colonisation by P.jiroveci in HIV negative patients with CD4+ counts of <400 cells/ mm^3 ^[39].

Kadoya et al [37] in their study on the occurrence of PCP in 75 patients with inflammatory myopathy and SLE, noted a significant association between radiological interstitial pulmonary fibrosis (IPF) and the risk of PCP (8.8% IPF in non-PCP vs 100% IPF in PCP, p < 0.001). In contrast, PCP has only rarely been reported in idiopathic pulmonary fibrosis [15], indicating that more than systemic steroids and pulmonary fibrosis are required to put patients at excessive risk of PCP.

### Prevention

Co-trimoxazole is commonly used for PCP prophylaxis in Wegener's granulomatosis when CD4+ counts are <300 cells/mm^3 ^[15] or even with normal counts in some centres [21]. This combination of antibiotics has been shown to be effective prophylaxis when used daily or thrice weekly at a dose of 960 mg in HIV positive patients [40]. Adverse effects occur in less than 20% of patients, usually manifesting as a rash, which resolves on temporary discontinuation and often does not recur on re-challenge [41,42].

Apart from Wegener's granulomatosis, identifying patients with AID who are at risk of PCP has proved a challenge, as the overall incidence is low. Nevertheless it remains an important issue, as AID patients contribute a considerable proportion of cases of PCP in HIV negative patients (up to 36%) and have a particularly poor prognosis, as discussed earlier.

Any method used to select patients for prophylactic treatment needs to be assessed against set standards and have a high sensitivity and specificity. The standards should address the percentage of PCP cases captured by the selection criteria (ideally 100% but in practice >80%) and the risk of the condition in the selected group (which should be significant). In HIV patients who meet the criteria for PCP prophylaxis as set out by the US Public Health Service [43], the annual risk of PCP is 18% [38]. As the mortality from PCP in HIV negative patients is approximately double that of HIV-positive patients [14-18,27], we would suggest that an annual risk >9% of PCP would be sufficient to justify prophylaxis.

In the study by Mansharamani et al, their proposed cut off of <300 CD4+ cells/mm^3 ^would capture 91% of cases of PCP in all HIV negative patients, but would also include 39–46% of patients on systemic steroids, most of whom would be unaffected by PCP. Administering prophylaxis to such large numbers of patients would unnecessarily expose patients to drug side-effects and potentially encourage drug resistance. However, analysis of their data reveals that the subgroup of patients with AID who developed PCP had CD4+ counts of <250 cells/mm^3 ^and six out of eight had counts <200 cells/mm^3 ^[19].

Given the laboratory costs, we would argue in favour of performing CD4+ counts after one month's immunosuppression only on patients who satisfy the following three screening criteria:

• Steroid dosage >15 mg prednisolone or equivalent/day

• >three months corticosteroid treatment proposed

• total lymphocyte count <600 cells/mm^3^

A CD4+ count <200 cells/mm^3 ^might then warrant the use of prophylactic co-trimoxazole, if the annual risk of PCP in these patients is greater than 9%. Most cases of PCP in patients with AID would be captured by these criteria, according to published series.

Clearly, further prospective investigation is required to gather sufficient data to validate any selection method. To justify our proposed threshold for prophylaxis we would need to know the risk of PCP for patients on steroid-based immunosuppression for AID with CD4+ counts of <200 cells/mm^3^, information which is currently unavailable.

## Summary

P. jiroveci infection in HIV negative patients presents more acutely and has a worse prognosis. The incidence of PCP in patients with AID is low, although these patients still represent a considerable proportion of all HIV negative cases. It is important to have a high index of suspicion of PCP when treating AID with steroid based immunosuppressive regimes, as early treatment could improve prognosis. The risk of infection is related to treatment with systemic steroid, ill-defined individual variation in steroid sensitivity and CD4+ lymphocyte count. Effective and relatively safe prophylaxis is available. Rather than opting for PCP prophylaxis on the basis of disease or treatment with cyclophosphamide, we argue the case for carrying out CD4+ lymphocyte counts on selected patients as a means of identifying individuals who are most likely to benefit from PCP prophylaxis. Further prospective trials are required to validate our proposed prevention strategy.

## Competing interests

The authors declare that they have no competing interests.

## Authors' contributions

AJ Carmichael initiated the discussion, appraised results, lead departmental debates and helped revise the manuscript. ES carried out the literature search, presented at meetings and wrote the original manuscript. All authors read and approved the final manuscript.

## Pre-publication history

The pre-publication history for this paper can be accessed here:


